# ProfNet, a method to derive profile-profile alignment scoring functions that improves the alignments of distantly related proteins

**DOI:** 10.1186/1471-2105-6-253

**Published:** 2005-10-14

**Authors:** Tomas Ohlson, Arne Elofsson

**Affiliations:** 1 Stockholm Blolnformatlcs Center, Stockholm University, SE-106 91 Stockholm, Sweden

## Abstract

**Background:**

Profile-profile methods have been used for some years now to detect and align homologous proteins. The best such methods use information from the background distribution of amino acids and substitution tables either when constructing the profiles or in the scoring. This makes the methods dependent on the quality and choice of substitution table as well as the construction of the profiles.

Here, we introduce a novel method called ProfNet that is used to derive a profile-profile scoring function.

The method optimizes the discrimination between scores of related and unrelated residues and it is fast and straightforward to use. This new method derives a scoring function that is mainly dependent on the actual alignment of residues from a training set, and it does not use any additional information about the background distribution.

**Results:**

It is shown that ProfNet improves the discrimination of related and unrelated residues. Further it can be used to improve the alignment of distantly related proteins.

**Conclusion:**

The best performance is obtained using superfamily related proteins in the training of ProfNet, and a classifier that is related to the distance between the structurally aligned residues. The main difference between the new scoring function and a traditional profile-profile scoring function is that conserved residues on average score higher with the new function.

## Background

Alignment of proteins is one of the fundamental methods in bioinformatics. Alignments are used to detect homology and to study evolutionary events. The ability to align distantly related proteins can be improved significantly by the inclusion of evolutionary information [1,2] or predicted features [3]. Although significant improvements of alignment qualities has been seen recently in CASP [4], it is not clear how much the improved performance is due to an improvement of alignment methodologies and how much is due to increased number of sequences and structures that can be used to span the distance between a query protein and a target structure. However, in a recent study we have shown that the average alignment quality, as measured by MaxSub [5], improved by 10% at the family level and 50% at the superfamily level by the use of profile-profile scoring instead of sequence-profile scoring [6]. These findings are comparable to the ones found in a number of recent studies [7-9].

Profile-profile alignments can be implemented in several different ways [10-16]. The fundamental difference between different profile-profile alignment methods lie in how they calculate the score between two profile vectors. A profile, as defined in this study, can be seen as a set of vectors where each vector contains the frequency of each amino acid at a particular position in a multiple sequence alignment. In traditional sequence-profile alignments the score is calculated by extracting (the log of) the probability for an amino acid in this vector. However, in profile-profile alignments it is necessary to compare two vectors and this can be done in several different ways, including; calculating the sum of pairs, the dot product or a correlation coefficient between the two vectors. In addition, information about the background frequency and substitution probabilities can be used. Although, it has been shown that profile-profile methods using a probabilistic model seem to be superior to other methods [6,8], it is quite likely that better profile-profile scoring functions could be developed.

Here we present ProfNet, a method to develop novel profile-profile scoring functions. ProfNet is based on the ability to separate related from unrelated residue pairs, and it uses an artificial neural network (ANN) trained to identify pairs of residues from structurally aligned proteins. We show that ProfNet provides significantly better identification of related residues than prob_score [17] and that it also can be used to provide a slight improvement of the alignment of distantly related proteins. Another advantage of this approach is that it makes it trivial to add additional information to the scoring function.

## Results

It could be expected that a good profile-profile scoring function should provide high scores if two profile vectors have similar amino acid distributions that differs from the background distribution. In addition, the score should include information about what amino acids are more likely to be exchanged with each other. In an earlier study we found that one profile-profile scoring method, prob_score [17], performed these tasks quite well [6]. However, it is quite possible that a better function could be found. In order to develop such a function we have develop the method ProfNet that separates residue pairs that should and should not be aligned. Here, it is assumed that residue pairs aligned in a structural alignment should also be aligned by the profile-profile scoring function, while residue pairs belonging to proteins from different folds should not be aligned at all. Therefore, the scoring function was trained to identify pairs of structurally aligned residues. Finally, the ability to correctly align protein pairs using this novel scoring function was tested.

### Identification of related residues

A set of artificial neural networks (ANNs) were trained to distinguish related, by structural alignments, and non-related residue pairs. The first set of networks were trained using a simple representation where all aligned residues were considered to be related and a set of unrelated residues were chosen from randomly selected positions in unrelated protein pairs. The ANNs were trained using different datasets containing proteins of varying degrees of similarity. The performance of the different ProfNet versions was measured using the Matthews correlation coefficient (MCC) and the number of standard deviations separating the related and non-related residue pairs, i.e. the Z-score. In table 1 it can be seen that the MCC values for prob_score drops from 0.51 for family to 0.17 for superfamily and to 0.13 for fold related scores. No large difference in performance between the identification of superfamily and fold related pairs can be found, indicating that the physiochemical aspects of protein similarity is of greatest importance at this level of similarity.

**Table 1:** MCC-values and the corresponding Z-scores for prob_score and ProfNet versions trained on different datasets. The ProfNet versions were trained on profile vector pairs from unrelated proteins and protein positions related at family (ProfNet_fam), superfamily (ProfNet_su), fold (ProfNet_fold), and all SCOP levels (family, superfamily and fold) (ProfNet_all). The training of ProfNet_S was done using superfamily related profile vector pairs as positive examples, and classified by the S-score instead of the binary classifiers used in the other cases. The results are shown for protein pairs related on family, superfamily and fold level. The best results are shown in bold.

	MCC			Z-score		
training	fam	su	fold	fam	su	fold
prob_score	**0.51**	0.17	0.13	1.53	0.69	0.35
ProfNet_fam	**0.51**	0.18	0.14	1.69	0.72	0.42
ProfNet_su	0.49	**0.19**	0.16	1.69	**0.81**	0.52
ProfNet_fold	0.26	0.12	0.13	0.89	0.51	0.47
ProfNet_all	0.50	0.18	0.16	**1.84**	**0.81**	0.50
ProfNet_S	0.45	0.18	**0.17**	1.58	0.79	**0.56**

**Figure 1:**
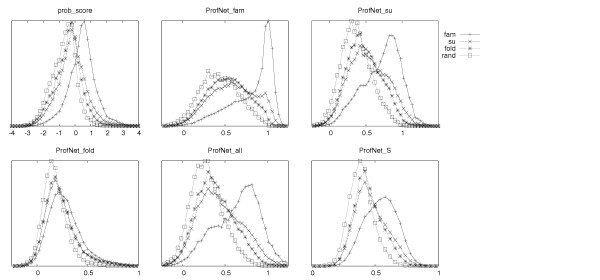
The distribution of scores from family, superfamily, fold related and randomly chosen profile vectors for prob_score and the five different ProfNet versions. The ProfNet versions were trained on profile vector pairs from unrelated proteins and proteins related at family (ProfNet_fam), superfamily (ProfNet_su), fold (ProfNet_fold), and a combination of family, superfamily and fold (ProfNet_all). The S-score training (ProfNet_S) was done using superfamily related vector pairs as positive examples, and classified by the S-score instead of the binary classifiers used in the other cases. All graphs show a Gaussian distribution, except for the family related scores in ProfNet_fam, which instead seems to follow an extreme-value like distribution. In each plot, the fraction of residues within a certain score range is plotted against the score. The exact values of the Y-axis have been left out for clarity.

For ProfNet_fam (which uses family related data in the training) a slight improvement over prob_score was seen. The Z-scores increased by 5–20% while the MCC values show a marginal increase at the superfamily and fold levels. In contrast to prob_score the ProfNet_fam scores for family related residues have a non-Gaussian distribution, see figure 1. For ProfNet_su (which uses superfamily related data in the training) the separation for distantly related residues got noticeable better at only a marginal lost performance at the family level, but when using only proteins from different superfamilies, but similar folds in the ANN training, (ProfNet_fold) the results are worse at all levels indicating that the evolutionary information is lost here. Using a combination of proteins from all SCOP levels (ProfNet_all) did perform similar to ProfNet_su, most likely because the superfamily set contain a set of residues related at a similar level. In figure 1 it can be seen that ProfNet_fold hardly separates the pairs at all, while all other scoring functions clearly separates the family-related, and to some extent also the superfamily-related from the non-related pairs.

In the above tests, all aligned positions were treated equally. However, certainly some of the aligned positions in the structural alignment are more closely aligned than others. Therefore, we also used a continuous function related to the distance between the two residues after the structural superposition. To measure the distance between two residues we used the S-score [18]. This ProfNet version, called ProfNet_S, performed quite well at the superfamily and fold levels, but did not distinguish the family related residues optimally. The Z-score for the fold related scores shows an improvement over prob_score by 60% and all the curves show a Gaussian like distribution, see Table 1 and Figure 1.

**Figure 2:**
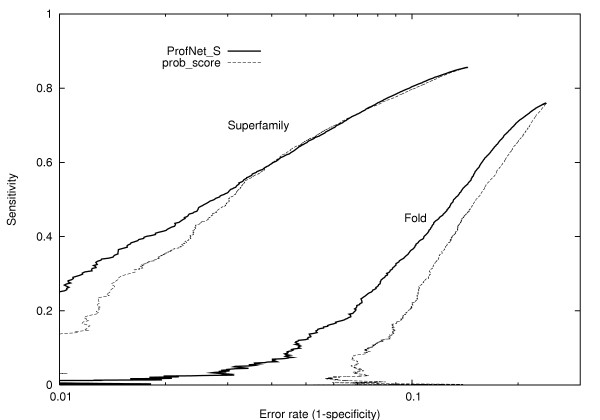
ROC plot based on the score for pairs of related and unrelated profile positions for prob_score and the S-score trained ProfNet. For each score the log of the error rate is plotted against the sensitivity for proteins related at superfamily, and fold level. The performance on family level was similar for the methods and was therefore left out for clarity.

A ROC-plot was constructed the same way as in Edgar 2004 [19] from the data used in the MCC analysis, figure 2. It can be seen that ProfNet_S is slightly better on superfamily level for low error rates, and clearly better at fold level.

### Alignment quality

**Figure 3:**
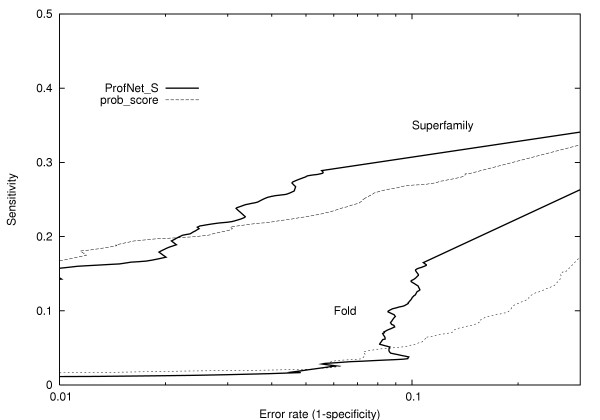
ROC plot based on protein model quality as measured by the MaxSub score for prob_score and the S-score trained ProfNet. For each score the log of the error rate is plotted against the sensitivity for proteins related at superfamily, and fold level. The performance on family level was similar for the methods and was therefore left out for clarity.

Although the identification of related residues might have some practical value [20], the real benefit from an improved scoring function would be if it could improve alignments and/or the detection of related proteins. It has earlier been shown that the alignment accuracy is increased by the use of profile-profile comparisons [7]. In an earlier study we noted a correlation between the ability to separate residues and the alignment quality if the gap-penalties were optimized for each scoring function individually [6]. In Table 2 it can be seen that the best ProfNet versions performed on par with prob_score on the ability to align proteins related on family or superfamily level, while a small (10%) increase in alignment qualities could be observed for the ProfNet_S and ProfNet_su versions for fold related proteins. The three ProfNet versions that provide the best alignments have the best identification of related residues. Taken this into account there seems to be some truth in our assumption that there should be a relationship between the ability to separate related from unrelated residues and aligning proteins. These results imply that some of the information needed to optimally align distantly related proteins are better captured by ProfNet than by prob_score. Furthermore, in figure 3, it can be seen that the ProfNet alignments produce more correct models at a given error rate than prob_score. A slightly improved performance can be seen for ProfNet on superfamily level for error rates > 0.03 and at error rates > 0.1, at the fold level.

**Table 2:** Alignment quality results for prob_score and the ProfNet versions trained on different datasets. The ProfNet versions were trained on profile vector pairs from unrelated proteins and protein positions related at family (ProfNet_fam), superfamily (ProfNet_su), fold (ProfNet_fold), and all SCOP levels (ProfNet_all). The training of ProfNet_S was done using superfamily related profile vector pairs as positive examples, and classified by the S-score instead of the binary classifiers used in the other cases. The average MaxSub scores are listed for a test sets with proteins related the family, superfamily or fold levels. The best results are shown in bold.

training	fam	su	fold
prob_score	0.56	**0.20**	0.063
ProfNet_fam	**0.57**	**0.20**	0.064
ProfNet_su	**0.57**	**0.20**	0.070
ProfNet_fold	0.55	0.17	0.057
ProfNet_all	**0.57**	**0.20**	0.067
ProfNet_S	**0.57**	**0.20**	**0.072**

Unfortunately, we did not see any significant improvement on the ability to detect related proteins using these novel scoring functions. The failure to increase fold recognition indicates that there still is work to do to find the optimal profile-profile scoring function. Quite likely, the construction of the negative training set was not done optimally.

## Discussion

Both prob_score and ProfNet provide a score for two profile vectors, that should be related to the similarity between the two (profile) positions. In the following sections we will compare these two functions, where ProfNet_S is used as a representative of the ProfNet method.

**Figure 4:**
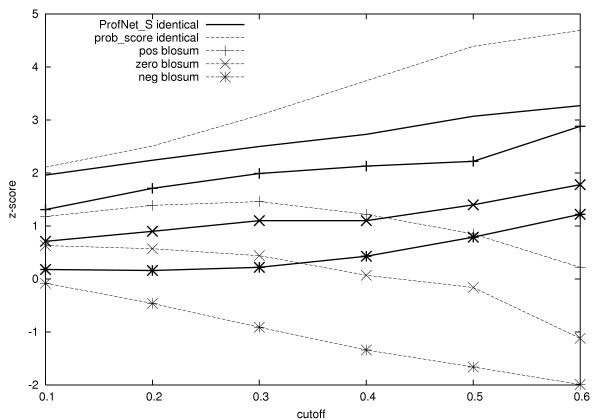
Average score for different classes of conserved residues. The classes were clustered by scores from vectors where the conserved residues in the vectors *i*) were identical, *ii*) had a positive BLOSUM62 score, *iii*) had a BLOSUM62 score of zero, and *iv*) had a negative BLOSUM62 score. The cutoff for the conserved residues are shown on the X-axis and the Z-score is shown on the Y-axis. A residue is considered conserved to a certain degree if it has a value in the profile vector above the cutoff. The solid bold lines are the scores for ProfNet_S, while the dotted lines are the scores for prob_score.

The correlation coefficient between prob_score and ProfNet is 0.68, indicating that the main features are similar but that there also exists differences. To understand the differences, the score from the scoring functions were examined for residues with varying degrees of conservation. The conservation was measured by the frequency of the most frequent amino acid in the profile vector. It should be noted that the frequency is not the directly observed frequency from the multiple sequence alignment but instead calculated from the PSI-BLAST profiles. Further, the residue pairs were sorted into four groups, pairs with identical conserved amino acids and pairs where the conserved amino acids in the two vectors had a positive, zero, or negative BLOSUM62 [21] score. In figure 4 it can be seen that for ProfNet the average scores for all groups increase with increased conservation, while for prob_score only the score for identical conserved residues increase. In table 3 the average score for the six groups, using a 30% conservation cutoff is shown. As expected both scoring functions score identical residues highest, while pairs of conserved unrelated residues score lower. However, it is notable that, on average, ProfNet provide higher scores than prob_score for all conserved pairs, regardless of the relationship between the two residues. ProfNet actually provides similar scores to a pair of conserved residues with negative BLOSUM scores as to one conserved and one non-conserved residue. Clearly, being conserved increases, for some reason, the chances to be structurally aligned.

**Table 3:** Average Z-scores for prob_score and ProfNet_S for the scores for different types of conserved residue pairs. ProfNet_S was trained on superfamily related profile vector pairs and using the S-score as a classifier. The pairs are grouped into pairs with identical residues, positive, zero and negative BLOSUM scores. Finally, the Z-scores for a pair containing one conserved and one non-conserved residue and two non-conserved residues are shown. The highest scores are shown in bold.

	prob_score	ProfNet_S
identical res	**3.09**	2.50
pos BLOSUM	1.46	**1.99**
zero BLOSUM	0.44	**1.10**
neg BLOSUM	-0.91	**0.22**
cons-non cons	**0.21**	0.15
non cons-non cons	**1.06**	0.53

**Figure 5:**
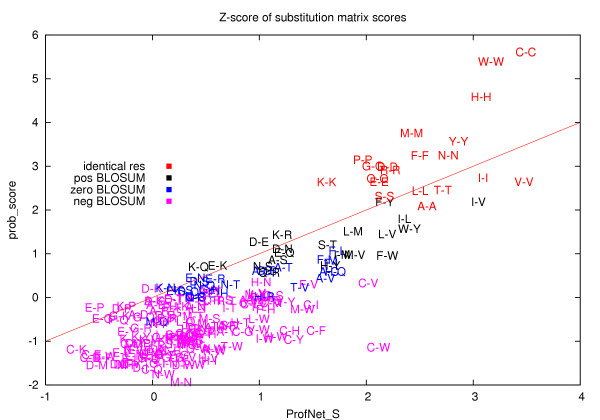
The Z-scores of the "substitution tables" generated by prob_score and ProfNet_S plotted against each other. The average Z-score for each residue pair is shown. The residues are written using their one-letter code.

To further investigate the differences in the scoring of conserved residues, substitution tables were derived from prob_score and ProfNet. The scores of the two tables were transformed into Z-scores and plotted against each other in figure 5. Here, it can be seen that prob_score ranks the residue pairs similar to BLOSUM62, giving the highest scores to identical pairs, while ProfNet on the other hand does not rank the residue pairs the same way. The correlation coefficient between the BLOSUM62 matrix and the scores from ProfNet was 0.75, compared to 0.95 for prob_score, see table 4. Figure 5 shows the same tendency that was observed in table 3, i.e. that ProfNet score most of the conserved residue pairs higher than prob_score. Clearly during the training of ProfNet other features than the BLOSUM62 classification has been learned. In figure 5 some outliers exist that might aid the explanation of the differences. ProfNet score pairs containing either a Cys or a Trp high while these pairs are scored low by prob_score. Trp and Cys are among the least frequent residues and a conservation of 30% (which is used as a cutoff for a conserved residue) might actually correspond to a higher degree of conservation than for a more common residue. Therefore, these scores could be explained by the general trend that the ProfNet scores increase with conservation. Another interesting outlier is the residue pair Ile-Val that is scored higher than many of the identical residue pairs by ProfNet. This indicates that the structural alignment might put more emphasize on physicochemical similarity than an evolutionary similarity.

**Table 4:** Correlation between different substitution tables and the profile-profile scores. Three tables, BLOSUM62, GONNET and JTT, are derived from sequence alignments while SDM is a structure based table. Three tables derived from ProfNet are also included, using the all (trained on data from all SCOP levels), su (trained on superfamily related data) and S-score (trained on superfamily related data and using the S-score as a classifier instead of a binary classifier) versions. STRUCTAL is a substitution table constructed from the residue matches found in the structurally aligned superfamily-related training set. The highest correlations are shown in bold.

subst. table	prob_score	ProfNet_S
BLOSUM62	**0.95**	0.75
GONNET	**0.93**	0.75
JTT	**0.87**	0.70
SDM	**0.89**	0.73

STRUCTAL	**0.85**	0.82

ProfNet_su	0.83	**0.96**
ProfNet_all	0.83	**0.95**
ProfNet_S	0.80	**1**

The scores from the substitution tables GONNET [22], JTT [23] and SDM [24] were also compared with the ProfNet derived substitution table, see table 4. The first two substitution tables are based on sequence alignments, while SDM is a structurally derived substitution table, i.e. based on structural alignments. Overall, prob_score showed a higher correlation to the substitution tables than to ProfNet, and ProfNet showed higher correlation with prob_score than with the substitution tables. This shows that ProfNet capture some of the substitution table information and some of the conservation information used in prob_score. It can also be seen that prob_score and ProfNet show comparable correlation with a substitution table created directly from the structurally aligned superfamily-related dataset.

### Future development

Here, we have only used the most obvious information from the profiles, i.e. the frequencies in the profile vectors for the development of the scoring function. One possible advantage of the ProfNet method is that it is easy to include other types of information, such as gap-information and predicted features, into the scoring functions.

## Conclusion

A novel method, ProfNet, to derive a profile-profile scoring function is shown to improve the discrimination between related and unrelated residue residues pairs. Further, ProfNet can be used to marginally improve the alignment quality of proteins related at the fold level. One benefit of this method is that it is easy to use and fast to evaluate, while one drawback is that a good and well balanced training set has to be used, and it is slower than prob_score. When choosing the training set, it seems as if the family related set is too focused on sequence similarity while the fold related training set on the other hand does not seem to include enough closely related pairs. The superfamily related training set could be seen as an intermediate, where the network will learn the features in the residue pairs that are essential when scoring unseen residue pairs. It was also found that using a binary classifier is not the best way to classify the training data, but instead some continuous classifier could be used. When using the superfamily related training data and ProfNet_S we see an improvement over prob_score by 31% in MCC (60% in Z-score) and 14% in average alignment quality for the fold related proteins. Interestingly, ProfNet clearly scores all conserved residues higher than prob_score does.

## Methods

### Profiles

We used the log-odds profiles obtained after ten iterations of PSI-BLAST [25] version 2.2.2, using an E-value cutoff of 10^-3 ^and all other parameters at default settings. The search was performed against nrdb90 from EBI [26]. The frequency profiles, used in prob_score, were back-calculated from the log-odds profiles obtained from PSI-BLAST as in [6]. The profiles used in ProfNet were created by a transformation of the log-odds profiles using a simple transformation as in PSI-PRED [27], trans formed_score(x)=1(1+exp−x)
 MathType@MTEF@5@5@+=feaafiart1ev1aaatCvAUfKttLearuWrP9MDH5MBPbIqV92AaeXatLxBI9gBaebbnrfifHhDYfgasaacH8akY=wiFfYdH8Gipec8Eeeu0xXdbba9frFj0=OqFfea0dXdd9vqai=hGuQ8kuc9pgc9s8qqaq=dirpe0xb9q8qiLsFr0=vr0=vr0dc8meaabaqaciaacaGaaeqabaqabeGadaaakeaacqWG0baDcqWGYbGCcqWGHbqycqWGUbGBcqWGZbWCcaaMc8UaemOzayMaem4Ba8MaemOCaiNaemyBa0MaemyzauMaemizaqMaei4xa8Laem4CamNaem4yamMaem4Ba8MaemOCaiNaemyzauMaeiikaGIaemiEaGNaeiykaKIaeyypa0ZaaSaaaeaacqaIXaqmaeaacqGGOaakcqaIXaqmcqGHRaWkieGacqWFLbqzcqWF4baEcqWFWbaCdaahaaWcbeqaaiabgkHiTiabdIha4baakiabcMcaPaaaaaa@5515@, where *x *is the value from the log-odds profile. In this study a profile is a matrix of dimensions 20*xL*, where *L *is the length of the target or query sequence. The term "profile vector" also known as "profile column" is a 20 × 1 dimensional vector with values corresponding to the occurrence of each amino acid, as calculated from the PSI-BLAST log-odds profiles, in a certain position in the profile.

### Scoring of two profiles

The input to the ProfNet scoring function is two transformed profile vectors, see above. The score between two profiles was calculated by first filling the dynamic programming matrix using ProfNet as a scoring function. After the matrix is filled, standard dynamic programming is used, with affine gap penalties. The number of calculations for each cell in the dynamic programming matrix for ProfNet is *hn *× *in*, where *hn *= # hidden nodes in the ANN and *in *= # input nodes (= 2 × 20), typically 20 × 40. The number of calculations for each cell in the dynamic programming matrix for prob_score is 2 × *r *× (1 + *x*), where r = # residues in the alphabet (= 20), and *x *is the number of calculations for a logarithm, i.e. 2 × 20 × (1 + *x*). In our implementation ProfNet is almost three times slower than prob_score.

In Mittelman *et. al. *[17] it is shown that probabilistic scoring functions is significantly better than other scoring functions and in Wang & Dunbrack 2004 [8], it is stated that with optimized gap penalties, most scoring functions behave similarly to one another in alignment accuracy. Taking all this into account, we choose to use the probabilistic scoring function prob_score instead of for example COMPASS or PICASS03 [17], since it was used in our previous study, where it was shown to be one of the best methods.

### Training sets

A subset of SCOP [28] version 1.57, class a to e, where no two protein domains have more than 75% sequence identity was used in the training of the artificial neural networks. For the positive training examples, protein pairs were structurally aligned using STRUCTAL [29] and all pairs of residues within 3 Å separation were used, while a set of negative training examples was created from randomly selected residue pairs from proteins of different folds. For the positive and negative data sets no more than 15 aligned positions from the same protein pair were used.

In an attempt to clarify what dataset to use in the ANN training we used five different datasets. The datasets consist of pairs of profile vectors corresponding to the aligned residues between protein pairs from the same family, superfamily (where no two proteins came from the same family), fold (where no two proteins came from the same superfamily), and a combination of family, superfamily and fold as positive examples and using randomly chosen vector pairs from unrelated protein positions as negative examples. The ANNs were trained to classify the profile vector pairs as related or unrelated (0 or 1). We also trained an ANN with the superfamily related set as positive examples, and trained to classify the profile vector pair according to the S-score [18]S−score=11+rmsd2/5
 MathType@MTEF@5@5@+=feaafiart1ev1aaatCvAUfKttLearuWrP9MDH5MBPbIqV92AaeXatLxBI9gBaebbnrfifHhDYfgasaacH8akY=wiFfYdH8Gipec8Eeeu0xXdbba9frFj0=OqFfea0dXdd9vqai=hGuQ8kuc9pgc9s8qqaq=dirpe0xb9q8qiLsFr0=vr0=vr0dc8meaabaqaciaacaGaaeqabaqabeGadaaakeaacqWGtbWucqGHsislcqWGZbWCcqWGJbWycqWGVbWBcqWGYbGCcqWGLbqzcqGH9aqpdaWcaaqaaiabigdaXaqaaiabigdaXiabgUcaRiabdkhaYjabd2gaTjabdohaZjabdsgaKnaaCaaaleqabaGaeGOmaidaaOGaei4la8IaeGynaudaaaaa@421C@. The *rmsd *is calculated between the *C*_*α *_atoms of the aligned residues.

The ratio between the positive and negative examples was not adjusted, instead all examples were used in the training as this was shown to produce the best alignment quality results for the superfamily related training set (data not shown). The size of the datasets ranges from 20 000 examples for the fold related and the negative dataset to 100 000 for the S-score trained examples.

### Matthews correlation coefficient

When comparing how well a method can separate positive and negative examples, such as the scores for related and unrelated profile positions, Matthews Correlation coefficient [30] (MCC) is a useful fitness measure. MCC takes into account both over-prediction and under-prediction and imbalanced data sets. It is defined as, MCC=tp×tn−fn×fp(tn+fn)(tn+fp)(tp+fn)(tp+fp)
 MathType@MTEF@5@5@+=feaafiart1ev1aaatCvAUfKttLearuWrP9MDH5MBPbIqV92AaeXatLxBI9gBaebbnrfifHhDYfgasaacH8akY=wiFfYdH8Gipec8Eeeu0xXdbba9frFj0=OqFfea0dXdd9vqai=hGuQ8kuc9pgc9s8qqaq=dirpe0xb9q8qiLsFr0=vr0=vr0dc8meaabaqaciaacaGaaeqabaqabeGadaaakeaacqWGnbqtcqWGdbWqcqWGdbWqcqGH9aqpdaWcaaqaaiabdsha0jabdchaWjabgEna0kabdsha0jabd6gaUjabgkHiTiabdAgaMjabd6gaUjabgEna0kabdAgaMjabdchaWbqaamaakaaabaGaeiikaGIaemiDaqNaemOBa4Maey4kaSIaemOzayMaemOBa4MaeiykaKIaeiikaGIaemiDaqNaemOBa4Maey4kaSIaemOzayMaemiCaaNaeiykaKIaeiikaGIaemiDaqNaemiCaaNaey4kaSIaemOzayMaemOBa4MaeiykaKIaeiikaGIaemiDaqNaemiCaaNaey4kaSIaemOzayMaemiCaaNaeiykaKcaleqaaaaaaaa@6201@. True positives (*tp*) are correctly predicted related scores, true negatives (*tn*) are correctly predicted unrelated scores, false negatives (*fn*) wrongly predicted related scores and false positives (*fp*) wrongly predicted unrelated scores. The MCC score is in the interval (-1,1), where one shows a perfect separation, and zero is the expected value for random scores. Three subsets (family, superfamily, and fold level) of the SCOP version 1.57 dataset that were not used in the training were used to calculate the MCC-values for each method.

### Artificial neural network training

The artificial neural networks (ANNs) were trained on 80% of the dataset, where a protein is only present in either the training or the test set. The neural network package Netlab in MatLab was used for the ANN training [31,32]. A linear activation function was chosen, and the training was carried out using the scaled gradient algorithm. Given two residues that should be aligned according to the training data, the ANN functions extracted their respective residue vectors from the transformed PSI-BLAST profiles, see above. The training of the ANNs was done using a grid search over the number of hidden nodes and number of training cycles. After the initial grid search, the search procedure was tuned to the area that produced the best results. At least 49 sets of parameters were tested for each ANN. The ANN-based scoring functions were chosen by selecting the ANN with the highest MCC-value and the minimum number of training cycles and hidden nodes. In the next step the ANN were used for the alignment quality test. The ProfNet scoring functions were implemented into the Palign [1,33] package

In summary, the ANNs were trained to identify related and unrelated profile vectors. The ANN use two transformed profile vectors, as described above, as input. The network should output a high score if two vectors are related and a low score otherwise. The ANNs that use a binary classifier outputs a value in the range (-0.6, 1.7), and the ANN that use the continuous S-score as a classifier output scores in the range (0, 1). In a sense, the network is trying to find a function that best can explain and correlate the training examples, i.e. the 40 numbers from the two profile vectors, and their output values. For the S-score trained network, the training examples classification are related to the rmsd between the *C*_*α *_atoms of the two residues that are aligned. With this strategy, the ANN is trained to predict the distance between the two residues, and hence if they should be aligned or not.

### Alignment quality

This dataset was also constructed from the same subset of SCOP version 1.57, class a to e, where no two protein domains have more than 75% sequence identity. From this dataset we included no more than 5 proteins from the same superfamily and no more than one model per domain target, we used in total 799 family, 672 superfamily and 602 fold related protein pairs. Among the superfamily related proteins, no proteins from the same family were included, and among the fold related proteins, no proteins from the same superfamily were included.

Throughout this study, only local alignments were used. For each alignment we created a model of the query protein and compared the structure of this model with the correct structure. We used MaxSub [5] which finds the largest subset of *C*_*α *_atoms of a model that superimpose well over the experimental model. We only report the MaxSub score because we noted in our earlier study [6] that the results obtained using other methods, such as LGscore [18], were almost identical. The parameters for the best MaxSub scores on superfamily and fold level are not always the same, therefore we show the results for a choice of parameters with MaxSub scores reasonably high at all levels.

### Parameters

The gap- and *shift*-parameters has to be optimized to get a good performance in the alignment quality test. The *shift *value is added to the score, so that an average score is negative, and the gap-opening (GO) and gap-extension (GE) is used to penalize for including a gap in the sequence. The gap-parameters in the alignment quality test were optimized with the constraint that the gap-extension penalty should be 5 or 10% of the gap-opening penalty. Ideally other ratios should be tried as well but as this would take too long time and we found reasonably good results, using the GO/GE ratio described above we did not spend any more time on the optimization. In addition this ratio between GO and GE has been seen to perform well in many other scoring schemes such as PSI-BLAST and prob_score. By using this rule we only had to search two two-dimensional parameter landscapes. We searched a grid of G0 = (0.1,0.2...,1.5) and *shift *= (-0.5, -0.45,...,1.5) for the ProfNet methods, and G0 = (0.2,0.3...,3.5) and *shift *= (-0.5,-0.45,...,1.5) for prob_score. The set of parameters with the best MaxSub score was then chosen.

### ROC plot

In the two ROC-plots, the error rate is plotted against the sensitivity (= *tp/*(*tp + fn*)). In figure 2 the error rate and sensitivity was calculated from scores of related and unrelated profile positions, i.e. from the MCC analysis data. In figure 3, the alignment quality was calculated for the superfamily related set that was used in the alignment quality test and a negative dataset. The negative dataset consists of 1000 unrelated protein pairs from SCOP version 1.57, class a to e, where no two protein domains have more than 75% sequence identity.

### Substitution matrices

In the comparison of ProfNet and prob_score, a conserved residue was defined as a residue with "frequency" (calculated from the converted PSI-BLAST profiles) above a certain cutoff in the profile frequency vector. In a non-conserved vector, no residue has a frequency above 0.10. To analyze how the methods score conserved residues, the average score was calculated between the conserved residues related at superfamily level from the test set used in the MCC test. To make the comparison more straightforward, the scores were transformed into Z-scores according to *Z-score*(*x*) = (*x - μ*)/*σ*, where *μ* is the average score over many randomly chosen examples and *σ *is the standard deviation. From these scores, substitution table-like matrices were derived for the methods. All different ProfNet versions produced the same outliers (data not shown).

## Authors' contributions

Tomas Ohlson wrote the code for the analysis, designed the test set and performed all experiments. Arne Elofsson participated in the design of the study. Both authors collaborated in writing the manuscript.
